# Opening a 60-year time capsule: sequences of historical poliovirus cold variants shed a new light on a contemporary strain

**DOI:** 10.1093/ve/veae063

**Published:** 2024-07-29

**Authors:** Morgane Chesnais, Erika Bujaki, Typhaine Filhol, Vincent Caval, Marie-Line Joffret, Javier Martin, Nolwenn Jouvenet, Maël Bessaud

**Affiliations:** Institut Pasteur, Université de Paris Cité, CNRS UMR 3569, Virus sensing and signaling Unit, 28 rue du Dr Roux, Paris 75 015, France; Laboratoire associé au Centre national de référence pour les entérovirus & paréchovirus, 28 rue du Dr Roux, Paris 75 015, France; Division of Vaccines, National Institute for Biological Standards and Control, Medicines and Healthcare products Regulatory Agency, Blanche Lane, South Mimms, Potters Bar, Hertfordshire EN6 3QG, United Kingdom; Institut Pasteur, Université de Paris Cité, CNRS UMR 3569, Virus sensing and signaling Unit, 28 rue du Dr Roux, Paris 75 015, France; Institut Pasteur, Université de Paris Cité, CNRS UMR 3569, Virus sensing and signaling Unit, 28 rue du Dr Roux, Paris 75 015, France; Institut Pasteur, Université de Paris Cité, CNRS UMR 3569, Virus sensing and signaling Unit, 28 rue du Dr Roux, Paris 75 015, France; Laboratoire associé au Centre national de référence pour les entérovirus & paréchovirus, 28 rue du Dr Roux, Paris 75 015, France; Division of Vaccines, National Institute for Biological Standards and Control, Medicines and Healthcare products Regulatory Agency, Blanche Lane, South Mimms, Potters Bar, Hertfordshire EN6 3QG, United Kingdom; Institut Pasteur, Université de Paris Cité, CNRS UMR 3569, Virus sensing and signaling Unit, 28 rue du Dr Roux, Paris 75 015, France; Institut Pasteur, Université de Paris Cité, CNRS UMR 3569, Virus sensing and signaling Unit, 28 rue du Dr Roux, Paris 75 015, France; Laboratoire associé au Centre national de référence pour les entérovirus & paréchovirus, 28 rue du Dr Roux, Paris 75 015, France

**Keywords:** cold mutants, cold variants, containment, enterovirus, vaccine-derived polioviruses, polioviruses, Saukett

## Abstract

Polioviruses (PVs) are positive strand RNA viruses responsible for poliomyelitis. Many PVs have been isolated and phenotypically characterized in the 1940s–50s for the purpose of identifying attenuated strains that could be used as vaccine strains. Among these historical PVs, only few are genetically characterized. We report here the sequencing of four PV strains stored for more than 60 years in a sealed box. These PVs are cold variants that were selected by Albert Sabin based on their capacity to multiply at relatively low temperatures. Inoculation of permissive cells at 25°C showed that two of the four historical virus stocks still contained infectious particles. Both viruses reached titres that were higher at 25°C than at 37°C, thus demonstrating that they were genuine cold variants. We obtained sequences that span virtually all the genome for three out of the four strains; a short sequence that partly covers the 5ʹ untranslated region was recovered for the last one. Unexpectedly, the genome of one historical cold variant (which derives from PV-3 Glenn) displayed a very high nucleotide identity (above 95%) with that of a PV strain (PV-3 strain WIV14) sampled in China in 2014 and then classified as a highly evolved vaccine-derived PV. Our analyses made this hypothesis very unlikely and strongly suggested that Glenn and WIV14 shared a very recent common ancestor with one another. Some strains used to produce the inactivated polio vaccine were also very close to Glenn and WIV14 in the capsid-encoding region, but they had not been sequenced beyond the capsid. We therefore sequenced one of these strains, Saukett A, which was available in our collection. Saukett A and WIV14 featured an identity higher than 99% at the nucleotide level. This work provides original data on cold variants that were produced and studied decades ago. It also highlights that sequences of historical PV strains could be crucial to reliably characterize contemporary PVs in case of release from a natural reservoir or from a facility, which is of highest importance for the PV eradication program.

## Introduction

The three serotypes of poliovirus (PV) are the etiologic agents of poliomyelitis. They belong to the species *Enterovirus C* (Family *Picornaviridae*, Genus *Enterovirus*). Identified in 1909 and cultivated in cell cultures as early as 1949, PVs have been widely studied for decades to develop efficient vaccines against poliomyelitis (Sabin 1985). Beyond vaccine development, PVs constitute very convenient models that have been used to tackle a broad variety of topics: they are easy to grow in cell cultures, they produce virus stocks with high titres, their genome is very short and can be easily manipulated to conduct reverse genetics experiments (Pfeiffer and Rall 2017). The PV genome consists of a unique molecule of single stranded RNA of about 7400 nucleotides (nt) in length. It contains a large open reading frame (ORF) flanked by two untranslated regions (UTRs); this ORF encodes a polyprotein secondarily cleaved into four structural proteins (VP1 to VP4) that form the virus particles and a set of non-structural proteins involved in the virus life cycle.

Launched in 1988 and coordinated by the World Health Organization, the Global Polio Eradication Initiative (GPEI) aims to eradicate PVs. The GPEI has succeeded in drastically reducing the number of poliomyelitis cases worldwide and has managed to eradicate wild strains of two of the three serotypes. In order to mitigate the risk of the release of infectious PVs from facilities, the GPEI has elaborated an action plan for PV containment that encourages the reduction of the number of laboratories and facilities allowed to hold PV infectious materials and the limitation of virus stocks retained by each of them (Anonymous 2022). In this framework, our laboratories have engaged a process of sequencing and destroying PV infectious material that is not of major importance.

Amongst the PVs stored in the Institut Pasteur’s polio lab, we focused on strains stored for more than 60 years in a box that linked two eminent scientists to one another. The box was sent by Albert Sabin to André Lwoff. Albert Sabin developed the three attenuated strains (so-called Sabin strains) that composed the historical oral polio vaccine. André Lwoff is a virologist who was awarded a Nobel Prize in 1965 for his works on genetic control of enzyme and virus synthesis carried out at Institut Pasteur together with Jacques Monod and François Jacob. The box contained four PVs of historical interest. They are variants selected *in vitro* by Albert Sabin for their ability to multiply at low temperature (25°C) (Sabin 1961). These four variants derive from strains isolated and studied in the 1950s when scientists were extensively searching naturally occurring or laboratory-developed PV strains with low neurotropic activity that could be used as live vaccine strains. P2149 and P2226 are PV-1 strains isolated from stools of healthy non-contact children during a non-epidemic period in Louisiana (Sabin 1956, 1960); ‘P712, Ch2ab’ (referred as P712 hereafter) is the PV-2 attenuated strain (also known as Sabin 2 strain) that has been used for decades in the trivalent oral polio vaccine (Sabin and Boulger 1973) and was previously sequenced (Rezapkin et al. 1999); it derives from a virus isolated from faeces of a healthy child living in Louisiana. Glenn is a PV-3 strain isolated before 1956 from a healthy child during a nonepidemic period in Cincinnati (Sabin 1956, 1960). These four strains were evaluated as oral vaccine candidates by Albert Sabin in the 1950s, but P2149, P2226, and Glenn were not retained because they appeared insufficiently attenuated (Sabin 1956). Later, these strains were used by Albert Sabin as progenitors to select cold variants through serial passaging in cell cultures at temperatures that were progressively decreased from 33°C to 25°C (Sabin 1960, 1961). The phenotypic properties of the cold variants were studied *in vitro* (Sabin 1960, 1961) and their neurovirulence was assessed in monkeys (Sabin 1960, 1961). Their behaviour when administrated to children was also evaluated in tests carried out at a foundling hospital in New York City (Sabin 1960). The efficiency of the Glenn cold variant as a vaccine strain was assessed in a small clinical trial conducted in the 1960s (Barnes et al. 1967). To our knowledge, no genetic data were available for these cold variants.

This study began with the objective of sequencing the genome of these strains before destroying them. Despite the prolonged storage of the samples, two viruses (P2149 cold variant and P712 cold variant) established infection in cell cultures. After amplification by RT-PCR, the virus genomes were sequenced. The genetic sequences thus obtained were compared to those of other PVs and non-polio enteroviruses (EVs). This analysis revealed an unexpected close relationship between Glenn and a PV-3 strain isolated in 2014, which we subsequently identified as a close relative of one strain used to make the inactivated polio vaccine, Saukett A.

## Materials and methods

### Attempts of virus cultivation

Virus cultivation was attempted in a BSL-3 facility by using human rhabdomyosarcoma (RD) cells, which are widely used for PV isolation from clinical or environmental samples (Thorley and Roberts 2016). Cells were grown in Minimum Essential Medium Eagle (Sigma) supplemented with 2 mM Glutamine and 5% foetal bovine serum in 25-cm^2^ flasks. Subconfluent monolayers were inoculated with 100 µL of initial sample diluted in 400 µL of phosphate-buffered saline (PBS). After inoculation, the monolayers were incubated at 25°C or 37°C under 5% CO_2_ and microscopically checked for 7 days to detect the appearance of cytopathogenic effects. After 7 days, the flasks were frozen/defrosted three times, and the supernatants were collected and clarified by centrifugation. Fresh subconfluent RD monolayers were inoculated with 500 µL of the supernatant and incubated for 7 days.

### Temperature sensitivity

The temperature sensitivity phenotype of the viruses was evaluated by titrating the same virus stocks at 25.0°C and 37.0°C. Virus titres were evaluated in RD cells, by determining the number of 50% tissue culture infective dose units (TCID_50_) per mL following the WHO standard protocol (Dunn 2016).

### RNA extraction and detection by real-time RT-PCR

Viral RNA of the cold variants was extracted from the initial samples and from positive cell culture supernatants with the High Pure Viral RNA kit (Roche Diagnostics, Meylan, France) following the manufacturer protocol. RNA extracts were either immediately used for RT-PCR amplification or stored at −80°C for further analysis. The presence of PV genomes in the extracts was assessed by using a pan-EV Taqman assay already published (Monpoeho et al. 2000).

### Genome amplification and sequencing of the cold variants

PV genome amplification was attempted from RNA extracted directly from the content of the vials by using different sets of generic primers already described (Bessaud et al. 2008, 2016, Joffret et al. 2018); these primers target conserved genomic regions and allow the generation of large amplicons that can be sequenced using Illumina platform. Illumina raw data were assembled by using CLC Genomics Workbench 20. Gaps were bridged by using primers specifically designed to target both sides of the gaps; the corresponding amplicons were sequenced with the Sanger technique. The Illumina and Sanger contigs were assembled by using CLC Main Workbench 23 to get the final contigs.

RNA was also extracted from the passage 2’s supernatants of the two positive cell cultures. Virus genome was amplified with two primer pairs against EV-C (Joffret et al. 2018) and sequenced by Illumina technology.

### Sequencing of the strain Saukett A

The poliovirus Saukett A strain is a reference strain from the National Institute for Biological Standards and Controls (UK) originally received from Connaught (Canada) (Minor et al. 1982). The whole genome RT-PCR product from the Saukett A strain was sequenced by next-generation sequencing using the MinION nanopore platform as previously described (Klapsa et al. 2022). The genetic sequence thus obtained was submitted to GenBank as Saukett/A_NIBSC (accession number PP972258).

### Genetic analyses

PV sequences of interest were retrieved from GenBank. Nucleotide sequences were aligned using CLC Main Workbench 23 and phylogenetic trees were reconstructed using the MEGA X program (Kumar et al. 2018). The evolutionary history was inferred by using the Maximum Likelihood method and Tamura-Nei model. The tree with the highest log likelihood is shown. Initial tree(s) for the heuristic search were obtained automatically by applying Neighbour-Join and BioNJ algorithms to a matrix of pairwise distances estimated using the Tamura-Nei model, and then selecting the topology with superior log likelihood value. The trees are drawn to scale, with branch lengths measured in the number of substitutions per site.

Pairwise identities between sequences were determined by using the SimPlot software (Lole et al. 1999) with a 200 nt-wide window and a step size of 20 nt.

## Results

### Opening a box sealed for 60 years

**Figure 1.**
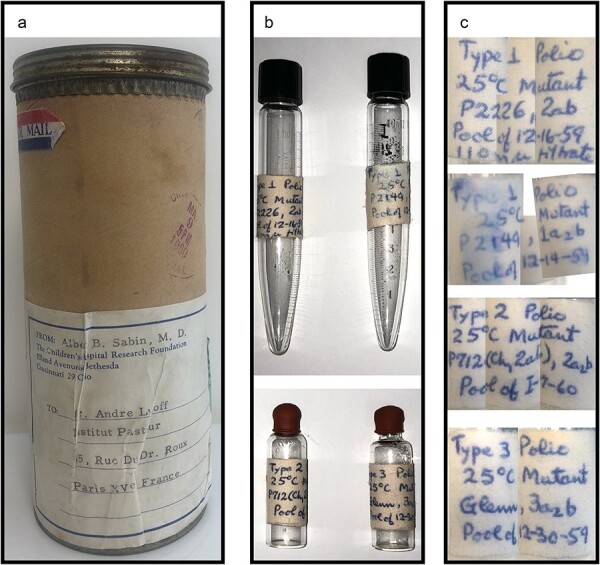
Photographs of the box sent by A. Sabin to A. Lwoff (a), of the four vials after their content has been transferred into other tubes (b), and of the vial labels (c).

The box consisted of a cardboard tube sealed by a metal cover with a postmark indicating the date of shipment: 9 May 1960 (Fig. 1a). It contained two 10-mL conic bottom glass vials and two smaller vials sealed by a rubber plug (Fig. 1b). The handwritten labels carried the full names of the original strains [‘PV-1 P2226, 2ab’; ‘PV-1 P2149, 1a_2_b’; ‘PV-2 P712 (Ch, 2ab), 2a_2_b’; ‘PV-3 Glenn, 3a_2_b’] and indicated that the vials contained variants adapted to growth at 25°C (Fig. 1c). The virus stocks were made in December 1959 and January 1960.

The PV-1 P2226 cold variant vial did not contain any liquid; its contents had apparently dried out, leaving a thin pink layer on the inner wall of the tube. To recover leftover biological materials (virus particles or viral RNA), the inner wall of this tube was washed with 300 µL of PBS prior to cell inoculation and RNA extraction. The three other vials contained around 4 mL each of red clear liquid that we assumed was clarified supernatant of cell culture inoculated with the virus indicated on the label.

### Two vials still contained infectious particles adapted to low temperatures

When RD cells were incubated at 37°C, no cytopathic effects (CPEs) were detected on cells inoculated with any of the four samples, even after two blind passages. By contrast, full lysis of the monolayers was observed after a second passage of P2149 and P712 cold variants in flasks incubated at 25°C, indicating that infectious virus particles survived the long-term storage. No CPEs were observed in flasks inoculated with P2226 and Glenn cold variants, suggesting that these two vials no longer contained infectious particles.

**Figure 2.**
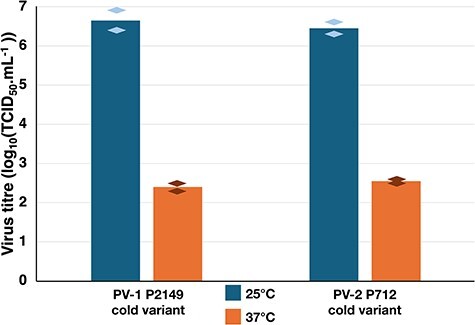
Temperature sensitivity of two viruses. The temperature sensitivity of the viruses was evaluated by titrating virus stocks at 25°C and 37°C on RD cells. The mean of two experiments is shown, with diamonds indicating the titre of each replicate.

The passage 2’s supernatants of P2149 and P712 cold variants grown at 25°C were titrated in parallel at 37°C and 25°C. Both reached a titre around 10^6.5^ TCID_50_.mL^−1^ when titration plates were incubated at 25°C and around 10^2.5^ TCID_50_.mL^−1^ when titration plates were incubated at 37°C (Fig. 2). These results demonstrated that the two viruses were genuine cold variants adapted for optimal growth at 25°C.

### Genetic analyses at nt positions known to modulate the virus phenotype

After RNA extraction performed on the RNAs extracted from the four vials, the real-time RT-PCR assay used to detect PV RNA gave positive results for all extracts, with Ct values ranging from 18.5 through 21.1. Amplification with a battery of generic and virus-specific primers followed by sequencing allowed the generation of sequences that spanned virtually the whole genome of three cold variants: P2149, P712, and Glenn (Table 1). For each of the two viruses that grew in cell cultures, the sequence generated from the historical vial and the sequence generated from the passage 2 supernatant were identical to each other. Despite the detection of P2226 RNA through real-time RT-PCR, we were only able to generate a very short sequence for this virus, probably because of the degradation of the RNA associated with the evaporation that took place in the corresponding vial. The 435 nt-long sequence thus obtained for P2226 partly covered the 5ʹUTR (Table 1).

**Table 1.** Characteristics of the contig obtained for each cold variant.

	Contig length (nt)	Beginning of the contig^a^	End of the contig^a^	GenBank accession number
PV-1 P2149	6923	73 (5ʹUTR)	7425 (3ʹUTR)	OR129532
PV-1 P2226	435	162 (5ʹUTR)	595 (5ʹUTR)	OR129533
PV-2 P712	7231	71 (5ʹUTR)	7426 (3ʹUTR)	OR129534
PV-3 Glenn	7254	162 (5ʹUTR)	7424 (3ʹUTR)	OR129531

a Nt position relative to PV-1 strain Mahoney genome numbering.

The P712 cold variant sequence was compared to that of the original strain [‘P712, Ch2ab’, also called Sabin 2, GenBank accession number AY124220 (Sabin and Boulger 1973, Rezapkin et al. 1999)] from which it was selected by serial passaging at decreasing temperatures (Sabin 1961). They differ from one another by only 7 nt differences that were evenly distributed along the genome, without any specific hotspot (Table 2). All differences were in the coding region and only one did not lead to an amino-acid substitution. The major determinant of the Sabin 2 attenuated phenotype (nt position 481 in the 5ʹUTR) is also involved in the temperature sensitivity of this strain (Macadam et al. 1991). It was unmodified in the corresponding cold variant, as was nt position 2909 (VP1-encoding region), which is the second determinant of the Sabin 2 attenuated phenotype (Macadam et al. 1993). The genomic sequences of the three strains from which P2149, P2226, and Glenn cold variants were selected are unknown. Therefore, we were not able to determine which nt positions were modified during the adaptation of these strains at low temperatures.

**Table 2.** Differences between the sequence of the original strain P712 and the sequence of the derived cold variant.

Protein	Nt position	Nt change	Aa position in the protein	Aa substitution
VP2	1264	G → A	104	A → T
VP3	2304	C → T	179	Synonymous
VP1	2501	A → T	7	E → V
	3365	A → G	301	E → G
2C	4414	G → A	98	A → T
	4456	G → A	112	V → I
3D	6190	G → A	69	D → N

Previous studies have shown that the Sabin 1 and 3 vaccine strains were less thermoresistant than the precursor strains from which they derive (PV-1 strain Mahoney and PV-3 strain Leon, respectively). Sabin 1 and Mahoney differ from each other by 55 nt changes (Nomoto et al. 1982). The P2149 cold variant genome was similar to Mahoney genome at 23 of these variable positions, similar to Sabin 1 at 14 positions and differed from both at 16 positions (Supplementary Table S1). Among these variable positions, seven have been experimentally proven to be involved in the temperature sensitivity of Sabin 1 (Tardy-Panit et al. 1993, Bouchard et al. 1995, Georgescu et al. 1995). At each of them, the nt found in the P2149 cold variant genome was similar to that found in Mahoney genome (Table 3).

**Table 3.** Comparison of Mahoney, Sabin 1, and P2149 cold variant sequences at nt positions that confer its temperature sensitivity to Sabin 1.

Region	Nt position	Mahoney	Sabin 1	P2149 cold variant
VP4	935	G	U	G
VP3	2438	U	A	U
VP1	2741	A	G	A
3D	6143	G	A	G
3D	6203	U	C	U
3D	7071	C	U	C
3ʹUTR	7441	–	G	Not sequenced

Sabin 3 and Leon differ from each other by 11 nt changes. The attenuation of Sabin 3 relies on three of them (nt positions 472, 2034, and 2493) (Cann et al. 1984), of which one (nt position 2034) is also the main determinant of the temperature sensitivity of this strain (Minor et al. 1989). The comparison of the genomes of Leon, Sabin 3, and Glenn cold variant revealed that the latter features nts that are similar to that of Leon genome at 8 out of 11 variable positions, including those responsible for the attenuated and temperature sensitive phenotype of Sabin 3 (Table 4).

**Table 4.** Comparison of Leon, Sabin 3, and Glenn cold variant sequences at nt positions that differentiate the attenuated vaccine strain Sabin 3 and the strain Leon from which it derives. Asterisks indicate nt positions that bear the Sabin 3 attenuation determinants.

Region	Nt position	Leon	Sabin 3	Glenn cold variant
5ʹUTR	220	G	U	A
5ʹUTR	472*	C	U	C
VP4	871	G	A	G
VP3	2034*	C	U	C
VP1	2493*	U	C	U
VP1	3333	A	G	A
2A	3464	A	G	G
2B	4064	U	C	U
3D	6061	C	U	C
3D	6127	U	C	G
3D	7165	G	A	G

### Genetic comparison of PV-1 cold variants with PV field strains

**Figure 3.**
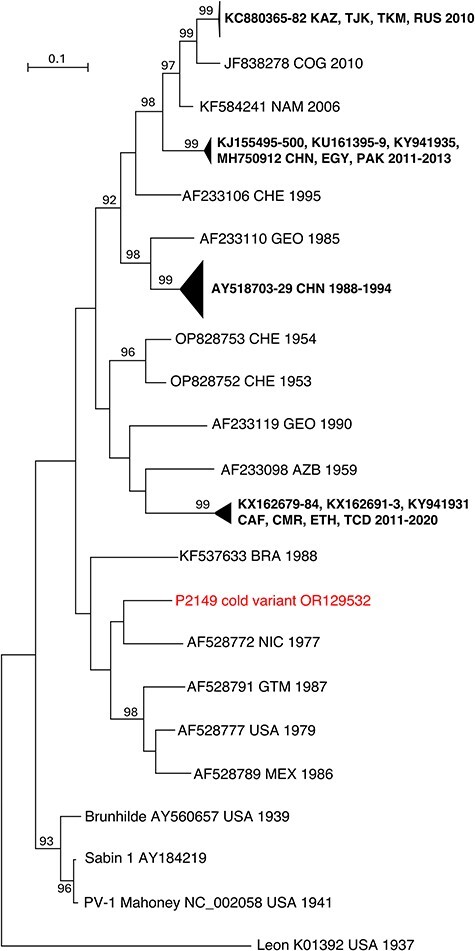
Genetic relationships of P2149 cold variant with Sabin 1 and all wild PV-1s available in GenBank, based on the VP1-encoding region. The sequence of PV-3 strain Leon was introduced as outgroup for rooting of the tree. Triangles represent lineages that have been collapsed for a better legibility of the trees; strains are labelled with their respective Genbank accession number followed by the country (ISO 3166-1 alpha-3 codes) and year of sampling, if known. Bootstrap values (1000 replicates) lower than 90% are not shown.

As the VP1 protein bears the major epitopes of the virus particles, PV strains are mainly characterized based on the VP1-encoding genomic region. In this region, PV-1 P2149 cold variant was relatively distant from other PV-1 strains isolated in the USA in the 1930s–1940s period, such as Mahoney or Brunhilde strains (Fig. 3); it was more closely related to strains sampled in the Americas in the 1970s and 1980s. The closest match found in public databases is a strain isolated in Nicaragua in 1977 (strain NIC77-8869, GenBank accession number AF528772) with featured 139 nt differences in the VP1-encoding sequence compared to P2149 cold variant. As no genetic data are available for NIC77-8869 outside this region, the genetic relationships between P2149 cold variant and NIC77-8869 in other genomic regions could not be studied.

Circulating PVs exchange genetic materials with one another and with some non-polio EVs members of the species EV-C: Coxsackievirus A11 (CVA11), CVA13, CVA17, CV20, CVA21, and EV-C99 (Combelas et al. 2011, Brouwer et al. 2020). To identify PVs or non-polio EVs featuring close relationships with the two PV-1 cold variants, a BLAST analysis was performed considering each of their genomic non-capsid regions. For both, the analysis did not reveal any strain with a high level of similarity: the closest match displayed a nt similarity <92%, whichever the genomic region (Table 5).

**Table 5.** Closest match identified in GenBank through BLAST analysis performed on each genomic region. NA: not applicable.

	PV-1 P2149 cold variant		PV-1 P2226 cold variant		PV-3 Glenn cold variant	
	Accession number	% similarity	Accession number	% similarity	Accession number	% similarity
5ʹUTR	DQ411556	91.32	AY928385	92.18	KY703697	95.17
2A	MF541373	85.68	NA	NA	KY703697	96.87
2B	AF465514	90.72	NA	NA	KY703697	96.91
2C	KU372652	91.19	NA	NA	KY703697	95.85
3A	KU372652	91.95	NA	NA	KY703697	94.64
3B	AF465514	88.06	NA	NA	KY703697	100.00
3C	KP247597	87.43	NA	NA	KY703697	94.54
3D	MG212444	88.07	NA	NA	KY703697	95.37

### Genetic comparison of Glenn cold variants with PV field strains

**Figure 4.**
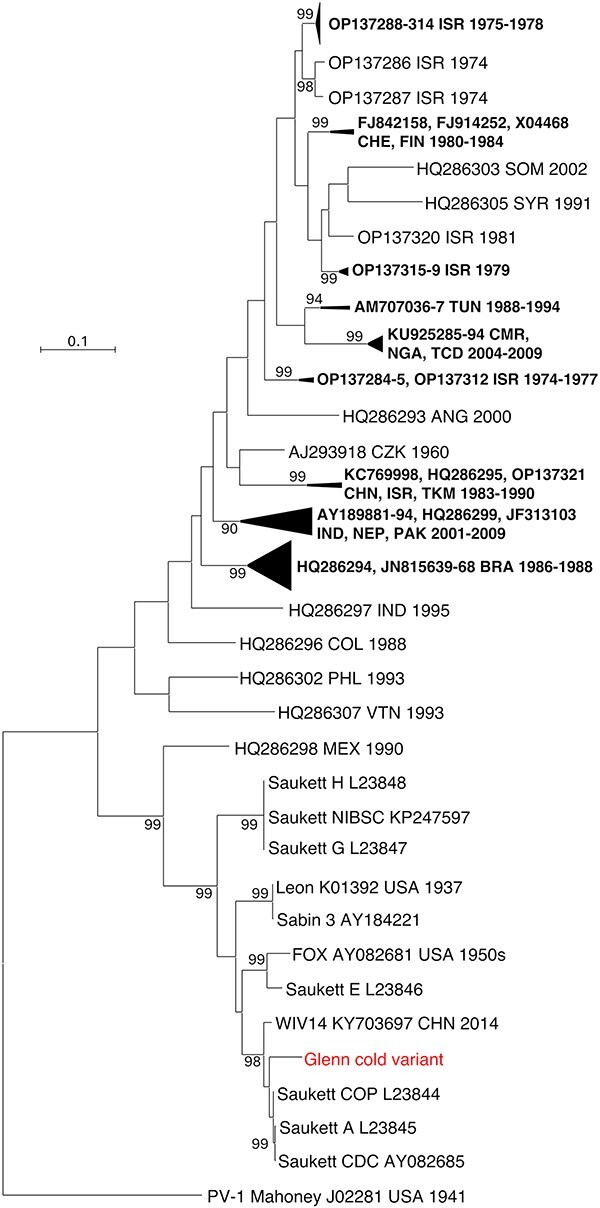
Genetic relationships of Glenn cold variant with Sabin 3 and all wild PV-3s available in GenBank, based on the VP1-encoding region. The sequence of PV-1 strain Mahoney was introduced as outgroup for rooting of the tree. Triangles represent lineages that have been collapsed for a better legibility of the trees; strains are labelled with their respective Genbank accession number followed by the country (ISO 3166-1 alpha-3 codes) and year of sampling, if known. Bootstrap values (1000 replicates) lower than 90% are not shown.

**Figure 5.**
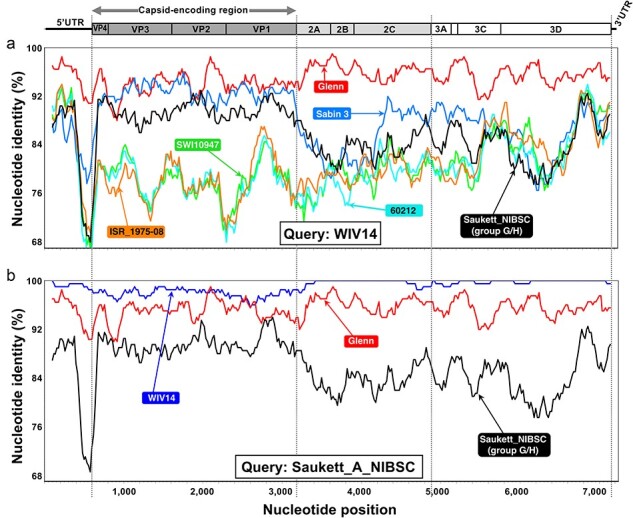
Pairwise comparison of the genome of PV-3 strain WIV14 (a) and PV-3 strain Saukett_A_NIBSC (b) with other PV-3 strains: Sabin 3, Saukett_NIBSC, 60 212, ISR_1975-08, SWI10947 (GenBank accession number AY184221, KP247597, FJ842158, OP137288, and FJ914252, respectively).

In the VP1-encoding region, Glenn cold variant fell into a branch made of strains that were isolated in the USA in the 1930s and 1950s and subsequently widely studied, such as Sabin 3, its precursor Leon (Sabin and Boulger 1973), and FOX (Koprowski 1957) (Fig. 4). This group also contained different strains identified as ‘Saukett’. These strains have been used to produce the injectable polio vaccine (IPV) after inactivation. All were supposed to derive from the same progenitor, P3/Saukett/USA/50, a strain isolated in 1950 in the USA. Nonetheless, previous studies revealed substantial antigenic and genetic differences between these strains and led to the hypothesis that they did not derive from the same progenitor (Minor et al. 1982, Ferguson et al. 1985). This hypothesis was subsequently reinforced by the sequencing of the capsid-encoding region of some of these strains, which revealed that they featured up to 304 nt differences to one another in the capsid (Kinnunen and Hovi 1989, Huovilainen et al. 1994). The different Saukett capsid sequences fall into three different phylogenetic groups (referred as Saukett A/COP/CDC, Saukett E, and Saukett G/H/NIBSC in Fig. 4) and probably derive from three distinct strains (Kinnunen and Hovi 1989, Huovilainen et al. 1994). In the capsid-encoding sequence, Glenn cold variant was closer to the Saukett A/COP/CDC strains than to the other Saukett strains (Fig. 4). The Glenn cold variant VP1-encoding sequence was also relatively close to that of WIV14 (GenBank accession number KY703697), a PV-3 strain sampled in 2014 from a patient during an outbreak of hand, foot, and mouth disease in China (Zhang et al. 2017). Because of its phylogenetic relationships with the live vaccine Sabin 3 strain, WIV14 was then identified as a highly evolved type 3 vaccine-derived poliovirus (Zhang et al. 2017). Our analysis revealed that the WIV14 VP1-encoding region is closer to that of Glenn cold variant (53 nt differences) than to that of Sabin 3 (69 nt differences). A BLAST analysis revealed that WIV14 is the closest match of Glenn, whichever the genomic region (Table 5). Overall, Glenn and WIV14 featured a nt identity higher than 95% to one another. Similarity plots showed that the nt identity between WIV14 and Sabin 3 dropped outside the capsid-encoding region while Glenn displayed with WIV14 an nt identity that remained evenly high throughout the genome (Fig. 5a). By contrast, WIV14 is genetically distant from Sabin 3 upstream and downstream the capsid region (82.30% and 82.26% nt identity, respectively). These observations suggested that WIV14 and Glenn cold variant shared a recent common ancestor with each other, which was unexpected for two strains isolated 60 years apart knowing the PV evolution rate in the capsid (Jorba et al. 2008) and the frequency of recombination events that continuously shuffle EV genomes (Kyriakopoulou et al. 2015, Muslin et al. 2019). WIV14 was also genetically close to the Saukett A/COP/CDC strains in the capsid region (97.84–97.95% nt identity) and less similar to Sabin 3 in this region (92.52% nt identity). None of the Saukett A, COP, or CDC strains had been sequenced beyond the capsid-encoding region, which made impossible genetic comparison in other genomic regions. Therefore, we sequenced the whole genome of the strain Saukett A available in the NIBSC’s collection. In the capsid-encoding region, the sequence we obtained was similar to that published 30 years ago (Huovilainen et al. 1994). The comparison of the genome of Saukett A with that of a representative of the Saukett G/H strains that was previously reported (GenBank accession number KP247597) (Mee et al. 2015) showed that the genetic divergence previously reported between the two strains in the capsid-encoding region (Huovilainen et al. 1994) was conserved across the whole genome (Fig. 5b) since the nt divergence was about 16% in both 5ʹUTR and non-structural region. The genomes of Saukett A and WIV14 differed from each other by only 70 nt differences (99% nt similarity across the genome). The repartition of the nt differences throughout the genome was very unbalanced since the capsid-encoding region gathered about 81% of them (Table 6). This result confirms that WIV14 does not derive from Sabin 3 but rather from a strain very close to Saukett A or from Saukett A itself.

**Table 6.** Nucleotide differences observed between the strains WIV14 and Saukett A.

Genomic region	Synonymous	Non-synonymous	Total
5ʹUTR	4	Not applicable	4
Capsid	47	10^a^	57
Non-structural proteins	6	3	9
3ʹUTR	0	Not applicable	0
**Total**	**57**	**13**	**70**

a Two non-synonymous mutations are in the same codon.

## Discussion

PVs have been intensively studied for decades and tens of PV strains isolated in the 1940s–1950s were phenotypically characterized using cell cultures and animal models. Yet, genetic data are very scarce for historical PV strains that were studied when genetic sequencing was not available. Apart from the most well-known PV strains, such as those used in the IPV or those the Sabin strains derived from, most of the genetic data available relate to PV strains sampled after the 2000s, of which most are Sabin-derived strains (Combelas et al. 2011, Burns et al. 2014). The sequencing of historical PV strains is of interest since it provides genetic data that can be used to reconstitute the epidemiology and the evolutionary trajectory of PVs. Furthermore, it contributes to build a virtual PV collection despite the destruction of the infectious stocks: since infectious PV particles can easily be rescued from synthetic RNAs, any PV strain could be revived if needed by using its genetic sequence. Retrospective genetic characterization of wild PV strains isolated before the sequencing era have been reported (Gavrilin et al. 2000, Jorba et al. 2008, Simonen et al. 2010, Tavares et al. 2013, Sanders et al. 2015, Shulman et al. 2022, Wildi et al. 2023) but, to our knowledge, no genetic data were available for the four cold variants sent by Albert Sabin to André Lwoff in 1960. These two scientists worked for years on PVs. Albert Sabin’s goal was mainly to get PV strains deprived of any neurovirulent properties that could be used as vaccine strains (Sabin 1956). For this purpose, he tested the neurovirulence of numerous PV variants in non-human primates (Horaud 1995). On their side, André Lwoff and his wife Marguerite Bourdaleix were interested in the role played by fever during virus infection and, in particular, how high body temperature could select virus variants with relatively high or low virulence (Lwoff 1959, 1969). In this context, they used PVs as models and showed a relation between the reproductive capacity of some PV strains at high temperatures and their neurovirulence (Lwoff and Lwoff 1959). From this discovery, both Sabin and Lwoff selected PV variants with various temperature optimums. While Lwoff was trying to understand how supra- or infra-optimal temperatures affect the virus cycle (Lwoff 1962), Sabin focused on the link between the reproductive capacity of PVs at different temperatures and neurovirulence. It is in this framework that he generated the four cold variants found in the Institut Pasteur’s collection (Sabin 1961).

The Institut Pasteur’s archives own several letters exchanged by Sabin and Lwoff that relate on their respective works on PVs. One of them, from Sabin, refers to the development of poliovirus cold variants (Supplementary Fig. S1) but does not provide any clue to understand why Albert Sabin sent these four specific cold variants to André Lwoff. The samples investigated in this study were stored for more than 60 years with no record of the storage conditions before 2007. Despite the long-term storage, two vials still contained infectious particles. The number of remaining infectious particles was probably very low since two passages were necessary to observe CPEs on cell cultures. At passage 2, virus growth was only detected in cells incubated at 25°C and the difference observed between titrations performed at 25°C and at 37°C showed that the viruses were cold variants and not original field strains. The ratios measured for the two strains between the titres at low and high temperature (around 4.0) were slightly higher than the ones reported by Albert Sabin in the original publication that describes the selection of these variants (around 3.0) (Sabin 1961). This discrepancy can be explained by the fact that the original work used 36°C as high temperature (rather than 37°C in our experiments), and by unavoidable differences between the Sabin’s experimental conditions and ours regarding cell types, cell culture medium, vessels, etc.

More than 60 years after the Lwoff-Bourdaleix’ pioneering works, the basis of PV reproductive adaptation to peculiar temperatures is still unclear. Previous works have identified various mutations involved in such adaptation in coding and non-coding regions of the virus genome (Omata et al. 1986, Minor et al. 1989, Macadam et al. 1991, Tardy-Panit et al. 1993, Bouchard et al. 1995, Georgescu et al. 1995, Dove and Racaniello 1997, Paul et al. 2000, Sanders et al. 2016). It is believed that these mutations have combinatorial effects that lead to the shift of the optimal growth temperature by modifying the folding and/or the thermal stability of virus proteins and/or secondary structures of the virus genome (Sanders et al. 2016). The mutations are not restricted to a limited number of nt positions since they are not conserved amongst cold adapted variants. For instance, during the development of three cold-adapted variants from the PV-1 strain Brunenders (Sanders et al. 2016), 31 mutations were observed of which only 8 were shared by the three variants; none of them were at positions previously identified as responsible for the temperature sensitive phenotype of Sabin 1 (Bouchard et al. 1995, Georgescu et al. 1995). The fact that P712 and P2149 cold variants do not display any of the nucleotides involved in the temperature sensitive phenotype of the strains Sabin 2 and Sabin 1 is therefore not surprising and confirms that the adaptation of PVs to low temperatures can result from multiple independent sets of mutations. The study of cold variants selected at 25°C from the strain Mahoney revealed that even individual mutations within the 2C-encoding region can recapitulate the cold-adapted phenotype (Dove and Racaniello 1997). The low number of differences between the P712 cold variant and the original Sabin 2 strains is thus not surprising, especially given that two differences were in the 2C-encoding region.

The sequences of ancient PV strains are of interest since they are representative of the genetic diversity that existed in the ecosystem formed by the EV-Cs when they were circulating. Although the genomes of the cold variants slightly differ from those of the original field strains from which they derive, it can be assumed that the number of mutations between the sequences of the cold variants and those of their respective precursors is relatively low as observed for P712 cold variant in this study and in previous characterizations of other cold variants (Bouchard et al. 1995, Sanders et al. 2016). Therefore, the sequences of the cold variants obtained in this work probably constitute a reliable trace of some PV lineages that circulated in the USA in the 1940s–1950s. This is why the close phylogenetic relationships between the Glenn cold variant and strain WIV14 over the entire genome made the hypothesis of WIV14 deriving from Sabin 3 improbable: even though PVs are known to often recombine and exchange non-capsid sequences with other EV-Cs, it is unlikely that a Sabin 3 strain excreted in the 2000s or 2010s evolved randomly to generate a sequence that matches so closely in all genomic regions with the sequence of the Glenn cold variant whose precursor was isolated 60 years earlier. The sequencing of the whole genome of Saukett A confirmed that WIV14 did not derive from Sabin 3 since Saukett A and WIV14 share more than 99% nt similarity across the genome. Most of nt differences between Saukett A and WIV14 are in the capsid-encoding region. In the EV genomes, this region is more variable than the 5ʹUTR and the non-structural region, whose evolution is more constrained by the presence of crucial RNA secondary structures or of sequences that encode for enzymatic domains (Oberste 2008). Nonetheless, the high concentration of the nt differences between WIV14 and Saukett A in the capsid-encoding region while the rest of the genome is highly conserved does not resemble the result of the genetic drift observed when EVs circulate in humans. WIV14 could then be derived from a close parent of Saukett A that does not appear in public databases. It could also derive from a laboratory strain originating from Saukett A whose capsid would have been modified by mutagenesis or by passages in conditions that led to mutations in the capsid, such as the presence of neutralizing antibodies for instance. In absence or any additional data, it is impossible to favour any hypothesis. The origin of WIV14 is therefore unidentified. Two possibilities must be explored:

1. The child from whom the virus was isolated was not infected by WIV14. In this case, a cross-contamination occurred at some point between the collection of the stool sample and the inoculation of the cells. The contaminating strain would not be Saukett A itself (since 70 nt differences exist between WIV14 and Saukett A) but a strain very close to Saukett A.
2. The child was genuinely infected by WIV14. In this case, WIV14 would be the descendant of a PV strain of the 1950s released from a natural reservoir in which it had lain dormant for decades or from a facility. The hypothesis of a natural reservoir could for instance imply the permafrost that preserves the infectivity of viruses that can be revived even after a long storage (Houwenhuyse et al. 2018). Nonetheless, such scenario could apply to any PV strain that circulated in the past decades; it would be surprising that it specifically involved a strain close to strains handled by dozens of factories around the world to make the IPV. Therefore, the hypothesis of a leak from a facility cannot be ruled out. Until regulations regarding PV containment were enforced in the 2010s, PVs were widely used for multiple purposes, such as basic researches, educational activities, or validation of disinfectants (Oberste 2018). To our knowledge, no research works involving Saukett A were published in the 2010s but, as mentioned above, this strain is used to produce the IPV in many places. Leaks from IPV factories were already documented (Offit Paul 2005, Duizer et al. 2016, 2017, 2023, Jeannoël et al. 2020). One of these leaks occurred through the accidental infection of a worker while no incident had been observed in the factory (Duizer et al. 2023); it would have remained undetected if regular environmental surveillance was not conducted around the factory (Duizer et al. 2023). It is therefore possible that several undocumented PV leaks occurred in the past from facilities handling PVs, especially when PVs were considered as low-risk viruses due to the existence of vaccines. WIV14 could thus have emerged after a PV strain released anywhere in the world had circulated before being sampled in China.

Whichever the source of WIV14, it is noteworthy that a similar situation already occurred in the 2000s when circulating EV-A71 strains were genetically very close to the prototype strain BrCr that had been sampled more than 30 years before (Yu et al. 2010, Zhu et al. 2013, Bessaud et al. 2014). The origin of these strains has never been identified. As underlined by the relationships between the strains WIV14, Glenn, Saukett A, and Sabin 3, the capsid-encoding region may not be sufficient to ascertain the origin of a field PV strain while the 5ʹUTR and the non-structural regions of the genome can provide decisive information. The current algorithm of the Global Polio Lab Network for the characterization of field PV strains relies on the sequencing of the VP1-encoding region. Since wild PV strains of serotypes 2 and 3 are supposed not to circulate anymore, any PV-2 or PV-3 strains are now assumed to derive from Sabin 2 or Sabin 3. When the number of mutations between the Sabin reference strains and the field strains is relatively low in the VP1, the status of Sabin-derived strains is clear. But when this number is high, there is a risk of misidentifying a strain that does not derive from one of the Sabin strains but from another strain, as exemplified by WIV14. This is why we suggest that PV strains that are sampled nowadays and that feature a relatively high number of mutations with the corresponding Sabin strain should be sequenced beyond the capsid and compared to other PV strains. Such comparison could help in identifying field strains that are suspiciously related to lineages supposed to be extinct. Therefore, the genetic sequences of historical PV strains are not only a valuable legacy for the scientific community: they could also be crucial for the eradication polio program to flag circulating strains that do not derive from the Sabin strains and trace their origin. Therefore, laboratories that still retain collections of historical PVs should be encouraged to sequence them before their destruction. This can be done with minimal risk of PV reintroduction since, as shown in a previous study (Shulman et al. 2022) and in this one, near-whole genome sequencing can be achieved from PV archived material with no need to reamplify viruses in cell culture.

## Supplementary Material

veae063_Supp

## Data Availability

Nucleotide sequences were submitted to GenBank (Accession numbers OR129531-OR129534 and PP972258).
