# Supplementary material for: Opening a 60-year time capsule: sequences of historical poliovirus cold variants shed a new light on a contemporary strain
Source: Virus Evol. 2024 Jul 29;10(1):veae063. doi: 10.1093/ve/veae063 (PMC11336667; doi:10.1093/ve/veae063)

Supplementary Fig. 1. Letter from Albert Sabin received by André Lwoff. Credits:  Archives de l’Institut Pasteur (Fonds André Lwoff).


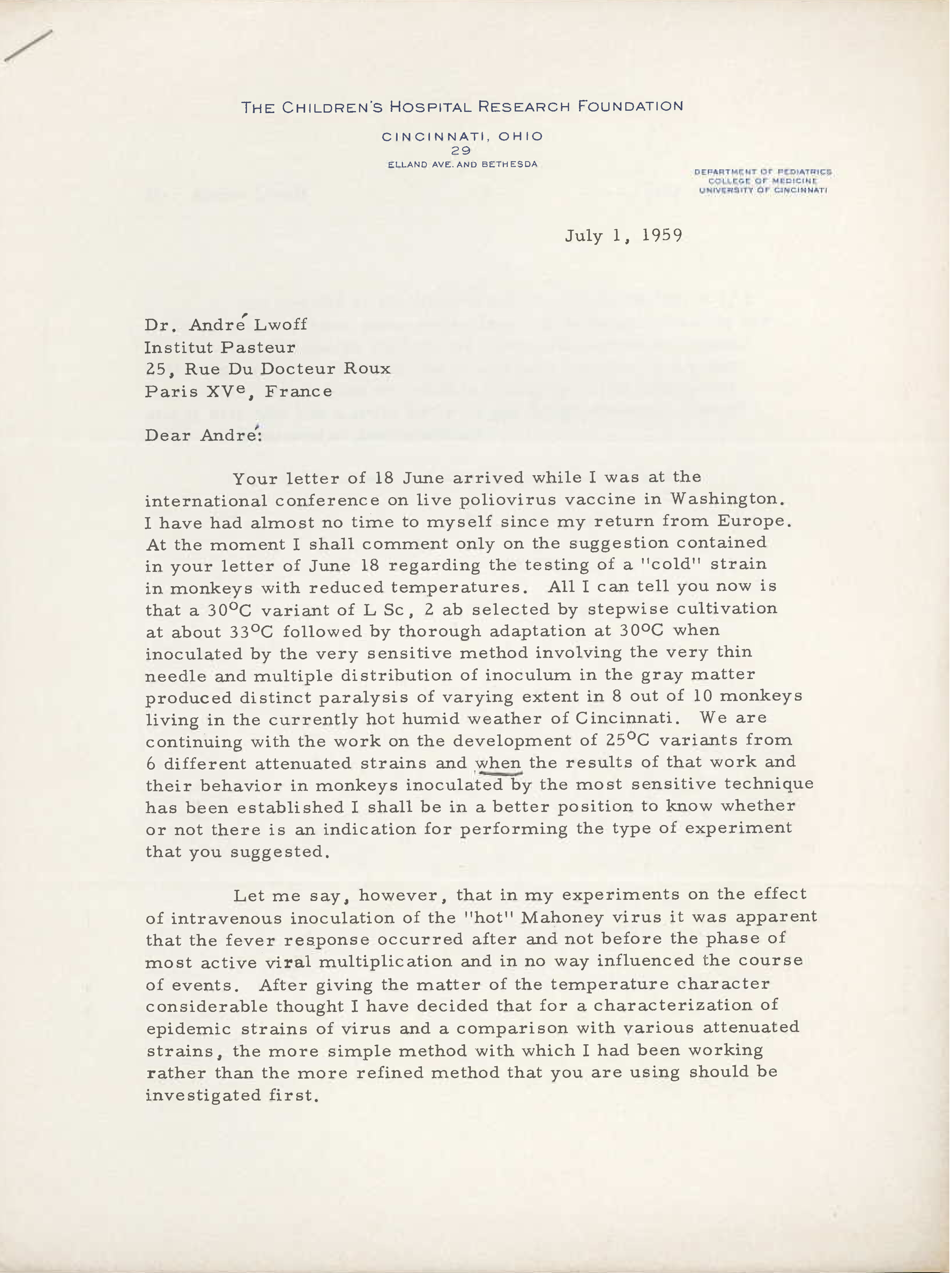


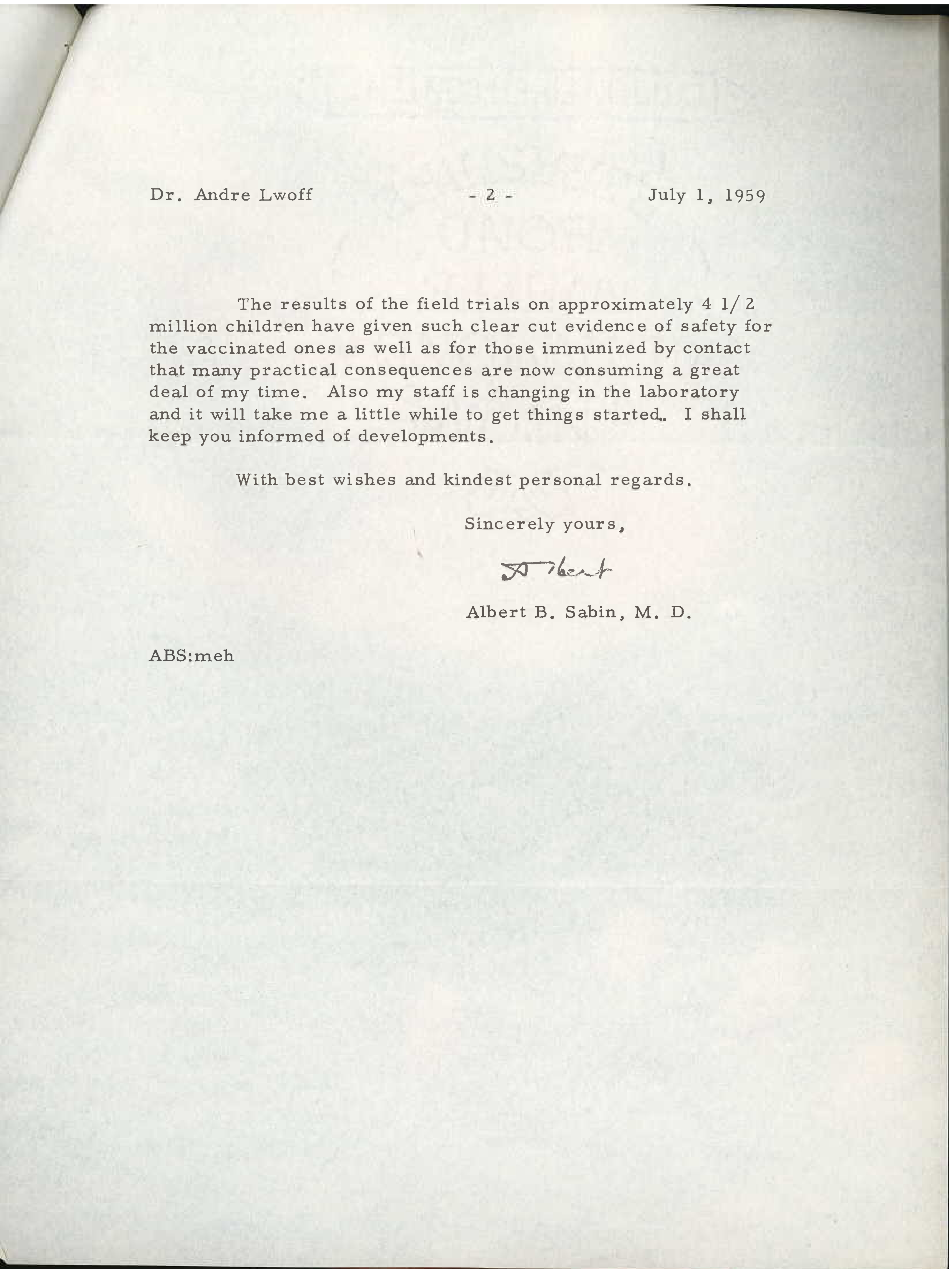

Supplement: veae063_Supp [file veae063_supp.zip › Supplementary Fig. 1.docx]
