# Supplementary material for: Opening a 60-year time capsule: sequences of historical poliovirus cold variants shed a new light on a contemporary strain
Source: Virus Evol. 2024 Jul 29;10(1):veae063. doi: 10.1093/ve/veae063 (PMC11336667; doi:10.1093/ve/veae063)
Supplement: veae063_Supp [file veae063_supp.zip › Supplementary Table 1.docx]

Supplementary Table 1. Comparison of Mahoney, Sabin 1 and P2149 cold variant sequences at nt positions that differentiate the attenuated vaccine strain Sabin 1 and the strain Mahoney from which it derives. Nt positions responsible for Sabin 1 attenuated phenotype are indicated by asterisk. Nt positions that confer its temperature sensitivity to Sabin 1 are in red. Positions shaded purple or green are those at which P2149 cold variant is similar to Mahoney or Sabin 1, respectively. n.s: not sequenced.

| **Nt position** | **Mahoney** | **Sabin 1** | **P2149 cold variant** |  | **Nt position** | **Mahoney** | **Sabin 1** | **P2149 cold variant** |
| --- | --- | --- | --- | --- | --- | --- | --- | --- |
| **5’UTR** |  |  |  |  | **2A** |  |  |  |
| 21 | U | C | n.s. |  | 3445 | C | U | C |
| 189 | C | U | U |  | 3460 | U | A | G |
| 480* | A | G | A |  | 3492 | G | A | A |
| 649 | C | U | G |  | 3766 | C | A | G |
| 674 | C | U | U |  | 3785 | U | A | U |
| **VP4** |  |  |  |  | **2B** |  |  |  |
| 935* | G | U | C |  | 3896 | A | G | G |
| 1208 | A | C | A |  | 3898 | C | A | A |
| 1228 | G | C | U |  | 3919 | C | U | C |
| 1442 | A | G | A |  | 4003 | C | U | U |
| 1465 | C | U | C |  | 4116 | U | C | U |
| 1490 | C | U | U |  | **2C** |  |  |  |
| 1507 | G | A | A |  | 4444 | U | C | C |
| 1747 | C | U | C |  | 4789 | A | G | C |
| **VP3** |  |  |  |  | 5107 | U | C | U |
| 1885 | A | U | C |  | **3A** |  |  |  |
| 1941 | C | A | U |  | 5137 | A | G | A |
| 1944 | C | A | C |  | **3B** |  |  |  |
| 2137 | U | C | C |  | 5420 | C | A | A |
| 2353 | U | C | U |  | **3C** |  |  |  |
| 2438* | U | A | U |  | 5440 | A | G | U |
| **VP1** |  |  |  |  | **3D** |  |  |  |
| 2545 | A | G | U |  | 6143 | G | A | G |
| 2585 | A | G | G |  | 6203* | U | C | U |
| 2741 | A | G | A |  | 6373 | C | U | C |
| 2749 | G | A | G |  | 6616 | G | A | U |
| 2762 | C | U | U |  | 6679 | U | C | C |
| 2775 | C | A | C |  | 6853 | C | U | C |
| 2795* | G | A | U |  | 7071* | C | U | C |
| 2879* | C | U | U |  | 7198 | U | A | C |
| 3163 | U | C | G |  | 7243 | U | A | A |
|  |  |  |  |  | 7410 | U | C | U |
|  |  |  |  |  | 7441 | - | G | n.s. |
